# High-pressure synthesis and crystal structure of SrGa_4_As_4_

**DOI:** 10.1107/S2056989019013562

**Published:** 2019-10-22

**Authors:** Valentin Weippert, Dirk Johrendt

**Affiliations:** a Ludwig-Maximilians-Universität München, Butenandtstrasse 5-13, D-81377 München, Germany

**Keywords:** crystal structure, strontium, gallium, arsenic, high-pressure synthesis

## Abstract

Strontium tetra­gallate(II,III) tetra­arsenide, SrGa_4_As_4_, was synthesized in a Walker-type multianvil apparatus under high-pressure/high-temperature conditions of 8 GPa and 1573 K. The com­pound crystallizes in a new structure type (*P*3_2_21, *Z* = 3) as a three-dimensional (3D) framework of corner-sharing SrAs_8_ quadratic anti­prisms with strontium situated on a twofold rotation axis (Wyckoff position 3*b*). This arrangement is surrounded by a 3D framework which can be described as alternately stacked layers of either condensed Ga^III^As_4_ tetra­hedra or honeycomb-like layers built up from distorted ethane-like Ga^II^_2_As_6_ units com­prising Ga—Ga bonds.

## Abstract 2

SrGa_4_As_4_ was synthesized under high-pressure/high-temperature conditions. The crystal structure com­prises a network of Ga^III^As_4_ tetra­hedra and ethane-like Ga^II^_2_As_6_ groups with Ga—Ga bonds.

## Chemical context   

The ternary systems *A*–*Tr*–As (*A* = Ca, Sr or Ba; *Tr* = Ga or In) contain numerous com­pounds with different crystal structures based on *Tr*As_4_ tetra­hedra which occur isolated (Kauzlarich & Kuromoto, 1991 ▸), as dimers, as chains (Stoyko *et al.*, 2015 ▸; He *et al.*, 2012 ▸), condensed to ethane-like *Tr*_2_As_6_ groups (Mathieu *et al.*, 2008 ▸; Goforth *et al.*, 2009 ▸; He *et al.*, 2011 ▸) or as large supertetra­hedral units (Weippert *et al.*, 2019 ▸). SrGa_4_As_4_ is the first high-pressure com­pound in this system and contains an unprecedented layer-like framework, thus expanding the structural variety of the *A*–*Tr*–As family.

## Structural commentary   

**Figure 1:**
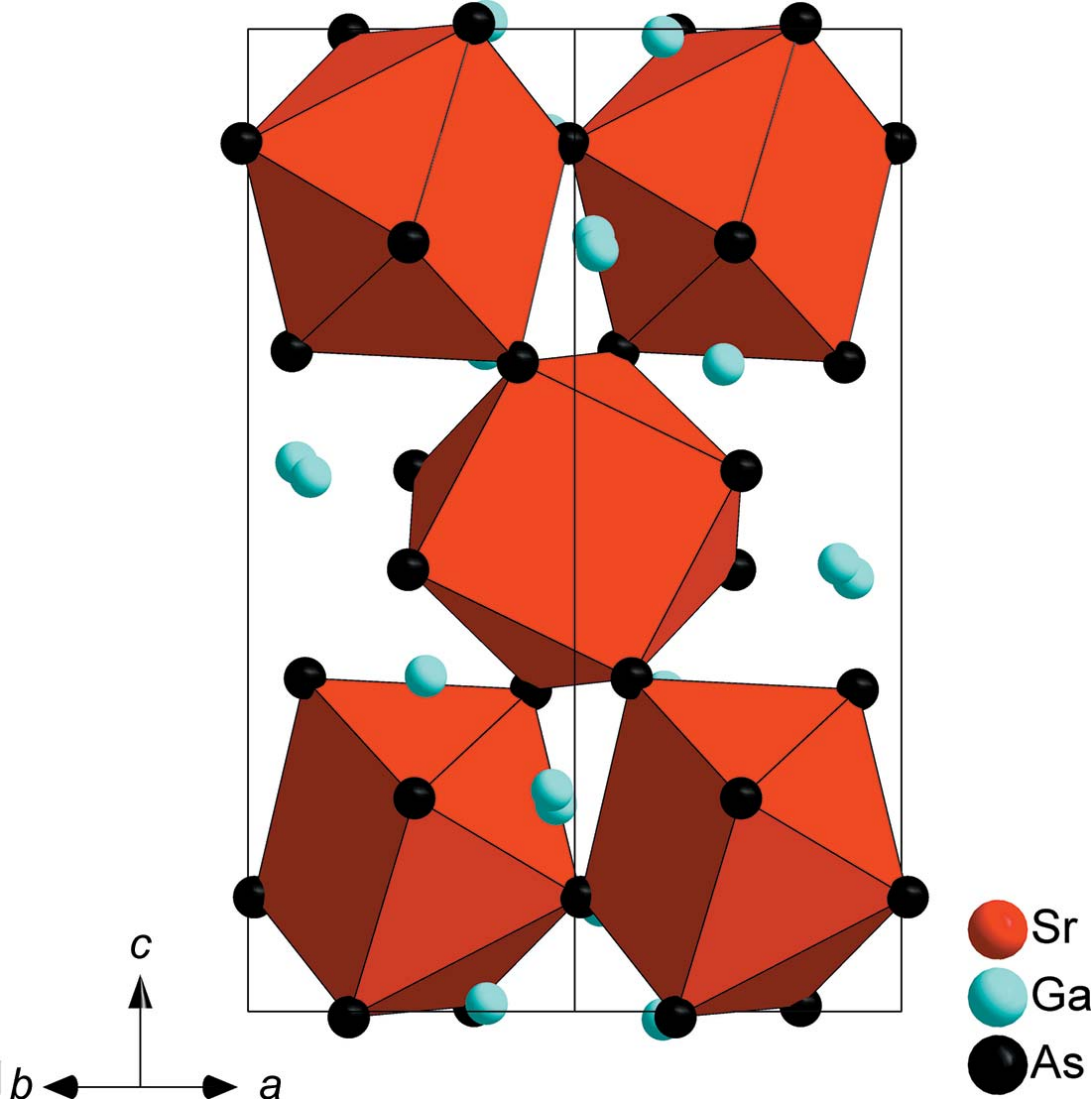
The unit cell of SrGa_4_As_4_, viewed along [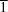 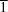
0], with the quadratic anti­prismatic strontium coordination spheres shown as red polyhedra.

**Figure 2:**
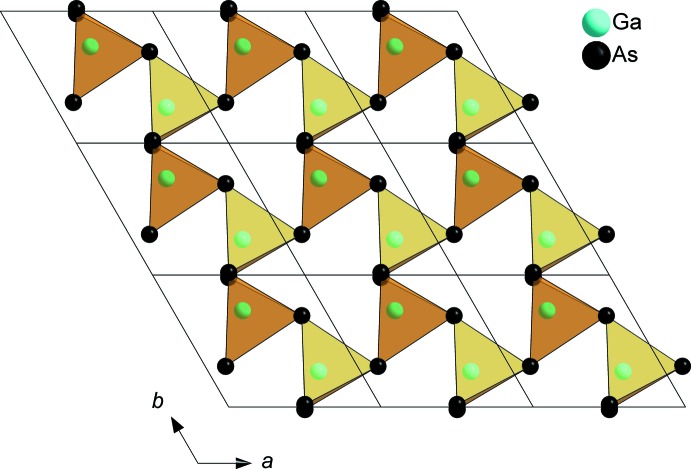
Edge- and corner-sharing GaAs_4_ tetra­hedra forming a layer with triangular voids viewed along [001].

**Figure 3:**
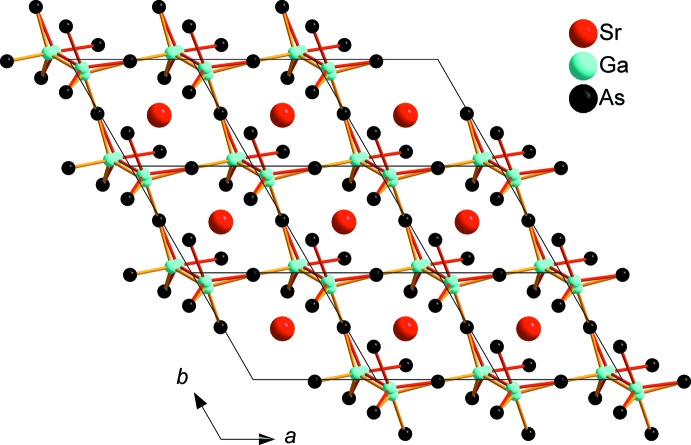
Corner-sharing Ga_2_As_6_ dumbbells with disordered Ga positions forming a honeycomb-like layer viewed along [001].

**Figure 4:**
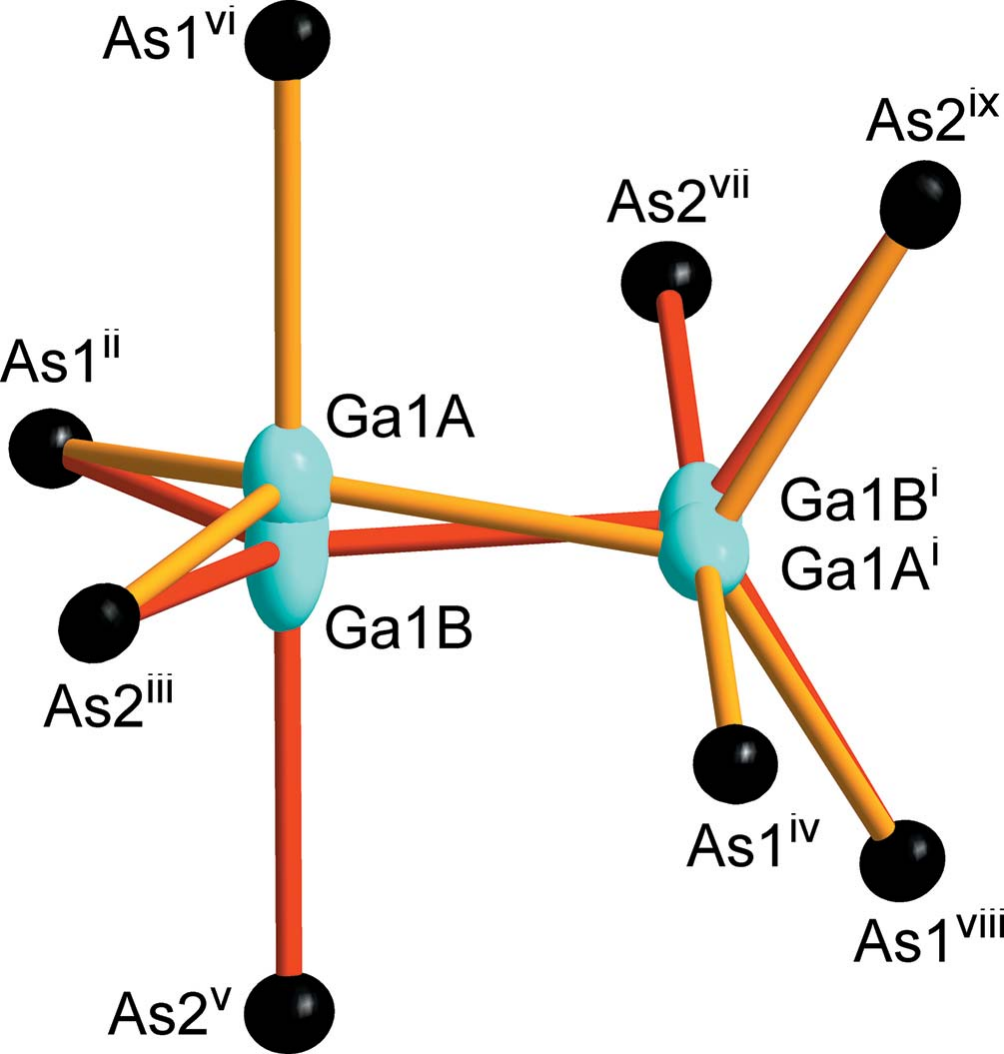
Ga_2_As_6_ groups with disordered Ga positions having an occupancy of 50%. Displacement ellipsoids are drawn at the 95% probability level. [Symmetry codes: (i) −*x* + 2, −*x* + *y* + 1, −*z* + 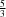; (ii) *y*, *x*, −*z* + 1; (iii) *x*, *y* + 1, *z* + 1; (iv) *y* + 1, *x* + 1, −*z* + 1; (v) *y* + 1, *x*, −*z* + 1; (vi) −*y* + 1, *x* − *y* + 1 *z* + 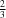; (vii) −*y* + 1, *x* − *y*, *z* + 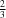; (viii) −*y* + 2, *x* − *y* + 1, *z* + 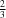; (ix) −*x* + 2, −*x* + *y* + 2, −*z* + 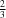.]

SrGa_4_As_4_ crystallizes in the space group *P*3_2_21 (No. 154) and constitutes a new structure type. Strontium is coordinated in a quadratic anti­prismatic manner by eight As atoms (Fig. 1 ▸). The anti­prisms are slightly distorted, with their quadratic planes twisted by ∼34° relative to each other instead of 45° for an ideal quadratic anti­prism. Sr—As distances range from 3.2665 (4) to 3.4560 (4) Å. The SrAs_8_ polyhedra are connected through common corners, each As atom shared by two quadratic anti­prisms, building up a three-dimensional (3D) framework. A similar structural motif is known for RbAg_2_SbS_4_, which crystallizes in the space group *P*3_1_21 (Schimek *et al.*, 1996 ▸). The surrounding construct in the two crystal structures differs however. SrGa_4_As_4_ contains a 3D Ga/As framework that can be subdivided into two types of layers with an *AB* stacking sequence along the *c* axis. The first type is built up from corner- and edge-sharing GaAs_4_ tetra­hedra forming sheets with triangular voids (Fig. 2 ▸). The tetra­hedra are distorted, with angles in the range of 100.790 (19)–127.996 (19)°, and have typical Ga—As distances of 2.4384 (5)–2.5470 (5) Å. The second layer type consists of distorted ethane-like Ga_2_As_6_ groups with nearly eclipsed conformations. The Ga_2_As_6_ groups are connected *via* common corners, forming a honeycomb-like sheet (Fig. 3 ▸). The Ga1*A* and Ga1*B* positions of the Ga–Ga dumbbell are disordered and were treated with split positions having an occupancy of 50% each (Fig. 4 ▸). The coordination of each of these Ga sites consists of three As atoms and one Ga atom forming trigonal pyramids, showing torsion angles of 114.5 (1)° for As1^vi^—Ga1*A*—Ga1*A* ^i^—As1^iv^ and 119.3 (1)° for As2^v^—Ga1*B*—Ga1*B* ^i^—As2^vii^ (for symmetry codes, see Fig. 4 ▸). The Ga—Ga distances range between 2.542 (8) and 2.572 (8) Å and are considered as Ga—Ga bonds, which is consistent with a charge-neutral com­pound. Ga—As distances between 2.477 (4) and 2.694 (2) Å for Ga1*A* are near to the covalent radii sum of 2.46 Å (Pauling, 1960 ▸). In com­parison, the trigonal pyramid around Ga1*B* is elongated, with Ga—As distances of 2.415 (4)–2.845 (2) Å.

## Synthesis and crystallization   

The starting material SrAs was synthesized by heating stoichiometric amounts of Sr (Sigma–Aldrich, 99.95%) and As (Alfa Aesar, 99.99999+%) in alumina crucibles, sealed in silica ampules under an atmosphere of purified argon for 20 h at 1223 K. The title com­pound was obtained *via* high-pressure synthesis using a modified Walker-type multianvil set-up driven by a 1000 t hydraulic press (Voggenreiter, Mainleus, Germany). A Cr_2_O_3_-substituted (6%) MgO octa­hedron (Ceramic Substrates & Components, Isle of Wight, UK) with an edge length of 18 mm, housing a ZrO_2_ sleeve with graphite sleeves (Schunk, Heuchelheim, Germany) for heating and a h-BN crucible (Henze, Kempten, Germany), was com­pressed with tungsten carbide cubes (Hawedia, Marklkofen, Germany) with an edge length of 11 mm. The starting materials SrAs (73.4 mg, 0.452 mmol), Ga (66.5 mg, 0.953 mmol, Alfa Aesar, 99.999%) and As (60.1 mg, 0.802 mmol) were mixed in a glove-box (H_2_O, O_2_ <1 ppm) and filled into the octa­hedron assembly. The reaction was carried out at 8 GPa and 1573 K, with a dwell time of 3 h. The temperature was increased and decreased over a period of 1 h. The assembly was opened in a glove-box, revealing crystals with a metallic luster.

The com­position of SrGa_4_As_4_ was verified by EDX measurements using a a Carl Zeiss EVO-MA 10 instrument with a Bruker Nano EDX detector. The experimental values [Sr 12 (1) at%, Ga 44 (2) at% and As 45 (1) at%] are in excellent agreement with the expected values (Sr 11.1 at%, Ga 44.4 at% and As 44.4 at%) within the typical error of the method, and confirm the com­position obtained from single-crystal X-ray diffraction data.

## Refinement   

Crystal data, data collection and structure refinement details are summarized in Table 1 ▸. The Ga1*A* and Ga1*B* positions were introduced as half-occupied split positions since one fully occupied position with a prolate ellipsoid caused residual densities in the order of 2.2 e Å^−3^. Upon exclusion of the Ga1*A*/Ga1*B* positions, the contour difference map in *PLATON* (Spek, 2009 ▸) shows two clearly separated maxima justifying this approach. Structural data were standardized with *STRUCTURE-TIDY* (Gelato & Parthé, 1987 ▸).

## Supplementary Material

Crystal structure: contains datablock(s) I, global. DOI: 10.1107/S2056989019013562/wm5523sup1.cif

CCDC references: 1957548, 1957548

Additional supporting information:  crystallographic information; 3D view; checkCIF report

## Figures and Tables

**Table 1:** Experimental details

Crystal data	
Chemical formula	SrGa_4_As_4_
*M*_r_	666.18
Crystal system, space group	Trigonal, *P*3_2_21
Temperature (K)	293
*a*, *c* (Å)	6.3615 (1), 16.5792 (2)
*V* (Å^3^)	581.05 (2)
*Z*	3
Radiation type	Mo *K*α
μ (mm^−1^)	37.42
Crystal size (mm)	0.10 × 0.05 × 0.05

Data collection	
Diffractometer	Bruker APEXII D8 Quest CCD
Absorption correction	Multi-scan (*SADABS*; Bruker, 2016 ▸)
*T*_min_, *T*_max_	0.446, 0.746
No. of measured, independent and observed [*I* > 2σ(*I*)] reflections	14966, 928, 918
*R*_int_	0.034
(sin θ/λ)_max_ (Å^−1^)	0.657

Refinement	
*R*[*F* ^2^ > 2σ(*F* ^2^)], *wR*(*F* ^2^), *S*	0.012, 0.025, 1.17
No. of reflections	928
No. of parameters	52
Δρ_max_, Δρ_min_ (e Å^−3^)	0.51, −0.69
Absolute structure	Flack *x* determined using 340 quotients [(*I* ^+^) − (*I* ^−^)]/[(*I* ^+^) + (*I* ^−^)] (Parsons *et al.*, 2013 ▸)
Absolute structure parameter	−0.024 (11)

Computer programs: *SAINT* (Bruker, 2016 ▸), *APEX3* (Bruker, 2016 ▸), *SUPERFLIP* (Palatinus & Chapuis, 2007 ▸), *EDMA* (Palatinus *et al.*, 2012 ▸), *SHELXL* (Sheldrick, 2015 ▸), *DIAMOND* (Brandenburg, 2014 ▸) and *PLATON* (Spek, 2009 ▸).

## supplementary crystallographic information

### Crystal data

**Table:**

SrGa_4_As_4_	*D*_x_ = 5.711 Mg m^−^^3^
*M**_r_* = 666.18	Mo *K*α radiation, λ = 0.71073 Å
Trigonal, *P*3_2_21	Cell parameters from 9912 reflections
*a* = 6.3615 (1) Å	θ = 3.7–30.4°
*c* = 16.5792 (2) Å	µ = 37.42 mm^−^^1^
*V* = 581.05 (2) Å^3^	*T* = 293 K
*Z* = 3	Block, black
*F*(000) = 882	0.10 × 0.05 × 0.05 mm

### Data collection

**Table:**

Bruker APEXII D8 Quest CCD diffractometer	928 independent reflections
Radiation source: Iµ S	918 reflections with *I* > 2σ(*I*)
Goebel Mirror monochromator	*R*_int_ = 0.034
combined φ and ω scans	θ_max_ = 27.9°, θ_min_ = 3.7°
Absorption correction: multi-scan (SADABS; Bruker, 2016)	*h* = −8→8
*T*_min_ = 0.446, *T*_max_ = 0.746	*k* = −8→8
14966 measured reflections	*l* = −21→21

### Refinement

**Table:**

Refinement on *F*^2^	Primary atom site location: iterative
Least-squares matrix: full	Secondary atom site location: difference Fourier map
*R*[*F*^2^ > 2σ(*F*^2^)] = 0.012	*w* = 1/[σ^2^(*F*_o_^2^) + (0.0102*P*)^2^ + 0.3943*P*] where *P* = (*F*_o_^2^ + 2*F*_c_^2^)/3
*wR*(*F*^2^) = 0.025	(Δ/σ)_max_ < 0.001
*S* = 1.17	Δρ_max_ = 0.51 e Å^−^^3^
928 reflections	Δρ_min_ = −0.69 e Å^−^^3^
52 parameters	Absolute structure: Flack *x* determined using 340 quotients [(I+)-(I-)]/[(I+)+(I-)] (Parsons *et al.*, 2013)
0 restraints	Absolute structure parameter: −0.024 (11)

### Special details

**Table:**

Geometry. All esds (except the esd in the dihedral angle between two l.s. planes) are estimated using the full covariance matrix. The cell esds are taken into account individually in the estimation of esds in distances, angles and torsion angles; correlations between esds in cell parameters are only used when they are defined by crystal symmetry. An approximate (isotropic) treatment of cell esds is used for estimating esds involving l.s. planes.

### Fractional atomic coordinates and isotropic or equivalent isotropic displacement parameters (Å^2^)

**Table:**

	*x*	*y*	*z*	*U*_iso_*/*U*_eq_	Occ. (<1)
Sr1	0.52374 (8)	0.000000	0.166667	0.01264 (11)	
Ga1A	0.8090 (7)	0.9427 (8)	0.8779 (2)	0.0120 (4)	0.5
Ga1B	0.8516 (7)	0.9303 (8)	0.8920 (2)	0.0175 (5)	0.5
Ga2	0.27470 (8)	0.54448 (7)	0.00800 (2)	0.00988 (9)	
As1	0.50915 (7)	0.49019 (6)	0.11634 (2)	0.00817 (8)	
As2	0.86593 (6)	0.17439 (6)	0.00577 (2)	0.00838 (8)	

### Atomic displacement parameters (Å^2^)

**Table:**

	*U*^11^	*U*^22^	*U*^33^	*U*^12^	*U*^13^	*U*^23^
Sr1	0.01406 (18)	0.0121 (2)	0.0111 (2)	0.00605 (12)	0.00104 (9)	0.00209 (17)
Ga1A	0.0136 (10)	0.0079 (6)	0.0131 (10)	0.0044 (6)	0.0032 (6)	0.0001 (6)
Ga1B	0.0184 (13)	0.0080 (7)	0.0206 (13)	0.0025 (7)	0.0107 (9)	−0.0018 (8)
Ga2	0.0105 (2)	0.00988 (18)	0.01119 (18)	0.00654 (16)	−0.00233 (16)	−0.00109 (14)
As1	0.00767 (16)	0.00801 (17)	0.00862 (16)	0.00376 (14)	−0.00032 (13)	0.00041 (12)
As2	0.00712 (16)	0.00890 (17)	0.00970 (17)	0.00444 (14)	0.00033 (13)	0.00055 (13)

### Geometric parameters (Å, º)

**Table:**

Sr1—As2	3.2665 (4)	Ga1A—As1^xii^	2.477 (4)
Sr1—As2^i^	3.2666 (4)	Ga1A—As2^xiii^	2.503 (4)
Sr1—As1^i^	3.2739 (4)	Ga1A—Ga1B^xiv^	2.5444 (13)
Sr1—As1	3.2739 (4)	Ga1A—Ga1A^xiv^	2.572 (8)
Sr1—As1^ii^	3.3048 (4)	Ga1A—As1^xv^	2.694 (2)
Sr1—As1^iii^	3.3048 (4)	Ga1B—As2^xiii^	2.415 (4)
Sr1—Ga1B^iv^	3.312 (4)	Ga1B—As1^xii^	2.515 (4)
Sr1—Ga1B^v^	3.312 (4)	Ga1B—Ga1B^xiv^	2.542 (8)
Sr1—Ga1A^vi^	3.346 (4)	Ga1B—As2^xvi^	2.845 (2)
Sr1—Ga1A^vii^	3.346 (4)	Ga2—As2^viii^	2.4384 (5)
Sr1—Ga2^viii^	3.3505 (4)	Ga2—As1	2.4668 (5)
Sr1—Ga2^ix^	3.3506 (4)	Ga2—As2^xvii^	2.4868 (5)
Sr1—As2^x^	3.4560 (4)	Ga2—As1^viii^	2.5470 (5)
Sr1—As2^xi^	3.4560 (4)	Ga2—Ga2^viii^	2.9844 (8)
			
As2—Sr1—As2^i^	120.45 (2)	Ga1A^xiv^—Ga1B—As2^xvi^	89.08 (12)
As2—Sr1—As1^i^	134.533 (8)	As2^xiii^—Ga1B—Sr1^xviii^	142.90 (10)
As2^i^—Sr1—As1^i^	78.453 (9)	As1^xii^—Ga1B—Sr1^xviii^	67.51 (9)
As2—Sr1—As1	78.453 (9)	Ga1B^xiv^—Ga1B—Sr1^xviii^	67.43 (7)
As2^i^—Sr1—As1	134.533 (8)	Ga1A^xiv^—Ga1B—Sr1^xviii^	72.45 (15)
As1^i^—Sr1—As1	119.39 (2)	As2^xvi^—Ga1B—Sr1^xviii^	63.55 (6)
As2—Sr1—As1^ii^	79.285 (11)	As2^xiii^—Ga1B—Sr1^xv^	67.96 (9)
As2^i^—Sr1—As1^ii^	74.128 (11)	As1^xii^—Ga1B—Sr1^xv^	137.34 (9)
As1^i^—Sr1—As1^ii^	66.217 (5)	Ga1B^xiv^—Ga1B—Sr1^xv^	68.95 (7)
As1—Sr1—As1^ii^	150.471 (11)	Ga1A^xiv^—Ga1B—Sr1^xv^	64.32 (16)
As2—Sr1—As1^iii^	74.128 (11)	As2^xvi^—Ga1B—Sr1^xv^	119.38 (13)
As2^i^—Sr1—As1^iii^	79.284 (11)	Sr1^xviii^—Ga1B—Sr1^xv^	136.38 (13)
As1^i^—Sr1—As1^iii^	150.470 (11)	As2^xiii^—Ga1B—Sr1^xix^	100.06 (10)
As1—Sr1—As1^iii^	66.217 (5)	As1^xii^—Ga1B—Sr1^xix^	39.58 (5)
As1^ii^—Sr1—As1^iii^	124.89 (2)	Ga1B^xiv^—Ga1B—Sr1^xix^	118.30 (16)
As2—Sr1—Ga1B^iv^	51.25 (5)	Ga1A^xiv^—Ga1B—Sr1^xix^	125.48 (7)
As2^i^—Sr1—Ga1B^iv^	73.07 (5)	As2^xvi^—Ga1B—Sr1^xix^	138.21 (12)
As1^i^—Sr1—Ga1B^iv^	109.71 (7)	Sr1^xviii^—Ga1B—Sr1^xix^	101.86 (10)
As1—Sr1—Ga1B^iv^	126.49 (6)	Sr1^xv^—Ga1B—Sr1^xix^	98.42 (5)
As1^ii^—Sr1—Ga1B^iv^	44.67 (7)	As2^xiii^—Ga1B—Sr1^xiii^	30.22 (6)
As1^iii^—Sr1—Ga1B^iv^	81.77 (7)	As1^xii^—Ga1B—Sr1^xiii^	86.93 (10)
As2—Sr1—Ga1B^v^	73.07 (5)	Ga1B^xiv^—Ga1B—Sr1^xiii^	155.24 (14)
As2^i^—Sr1—Ga1B^v^	51.25 (5)	Ga1A^xiv^—Ga1B—Sr1^xiii^	146.47 (15)
As1^i^—Sr1—Ga1B^v^	126.49 (6)	As2^xvi^—Ga1B—Sr1^xiii^	80.65 (8)
As1—Sr1—Ga1B^v^	109.71 (7)	Sr1^xviii^—Ga1B—Sr1^xiii^	128.07 (9)
As1^ii^—Sr1—Ga1B^v^	81.77 (7)	Sr1^xv^—Ga1B—Sr1^xiii^	93.16 (8)
As1^iii^—Sr1—Ga1B^v^	44.67 (7)	Sr1^xix^—Ga1B—Sr1^xiii^	80.08 (6)
Ga1B^iv^—Sr1—Ga1B^v^	45.14 (14)	As2^viii^—Ga2—As1	127.996 (19)
As2—Sr1—Ga1A^vi^	111.75 (6)	As2^viii^—Ga2—As2^xvii^	101.790 (19)
As2^i^—Sr1—Ga1A^vi^	123.12 (5)	As1—Ga2—As2^xvii^	107.308 (18)
As1^i^—Sr1—Ga1A^vi^	48.02 (5)	As2^viii^—Ga2—As1^viii^	112.107 (18)
As1—Sr1—Ga1A^vi^	74.76 (5)	As1—Ga2—As1^viii^	100.790 (19)
As1^ii^—Sr1—Ga1A^vi^	95.97 (6)	As2^xvii^—Ga2—As1^viii^	104.987 (18)
As1^iii^—Sr1—Ga1A^vi^	138.60 (6)	As2^viii^—Ga2—Ga2^viii^	160.190 (16)
Ga1B^iv^—Sr1—Ga1A^vi^	135.24 (5)	As1—Ga2—Ga2^viii^	54.718 (14)
Ga1B^v^—Sr1—Ga1A^vi^	174.30 (10)	As2^xvii^—Ga2—Ga2^viii^	94.821 (13)
As2—Sr1—Ga1A^vii^	123.12 (5)	As1^viii^—Ga2—Ga2^viii^	52.245 (14)
As2^i^—Sr1—Ga1A^vii^	111.75 (6)	As2^viii^—Ga2—Sr1^xx^	66.554 (14)
As1^i^—Sr1—Ga1A^vii^	74.76 (5)	As1—Ga2—Sr1^xx^	164.526 (19)
As1—Sr1—Ga1A^vii^	48.02 (5)	As2^xvii^—Ga2—Sr1^xx^	70.850 (14)
As1^ii^—Sr1—Ga1A^vii^	138.60 (6)	As1^viii^—Ga2—Sr1^xx^	65.805 (12)
As1^iii^—Sr1—Ga1A^vii^	95.97 (7)	Ga2^viii^—Ga2—Sr1^xx^	109.825 (18)
Ga1B^iv^—Sr1—Ga1A^vii^	174.30 (10)	As2^viii^—Ga2—Sr1^xxi^	65.922 (13)
Ga1B^v^—Sr1—Ga1A^vii^	135.23 (5)	As1—Ga2—Sr1^xxi^	62.098 (13)
Ga1A^vi^—Sr1—Ga1A^vii^	45.20 (14)	As2^xvii^—Ga2—Sr1^xxi^	126.697 (18)
As2—Sr1—Ga2^viii^	43.223 (9)	As1^viii^—Ga2—Sr1^xxi^	128.043 (18)
As2^i^—Sr1—Ga2^viii^	161.548 (17)	Ga2^viii^—Ga2—Sr1^xxi^	112.066 (16)
As1^i^—Sr1—Ga2^viii^	118.760 (14)	Sr1^xx^—Ga2—Sr1^xxi^	131.844 (12)
As1—Sr1—Ga2^viii^	45.205 (10)	As2^viii^—Ga2—Sr1^xvii^	94.008 (14)
As1^ii^—Sr1—Ga2^viii^	105.550 (10)	As1—Ga2—Sr1^xvii^	87.434 (14)
As1^iii^—Sr1—Ga2^viii^	86.374 (9)	As2^xvii^—Ga2—Sr1^xvii^	33.956 (10)
Ga1B^iv^—Sr1—Ga2^viii^	93.55 (5)	As1^viii^—Ga2—Sr1^xvii^	137.041 (15)
Ga1B^v^—Sr1—Ga2^viii^	110.30 (5)	Ga2^viii^—Ga2—Sr1^xvii^	105.802 (10)
Ga1A^vi^—Sr1—Ga2^viii^	75.33 (5)	Sr1^xx^—Ga2—Sr1^xvii^	97.358 (11)
Ga1A^vii^—Sr1—Ga2^viii^	81.05 (6)	Sr1^xxi^—Ga2—Sr1^xvii^	93.202 (9)
As2—Sr1—Ga2^ix^	161.548 (17)	As2^viii^—Ga2—Sr1	154.513 (14)
As2^i^—Sr1—Ga2^ix^	43.223 (9)	As1—Ga2—Sr1	29.480 (10)
As1^i^—Sr1—Ga2^ix^	45.205 (10)	As2^xvii^—Ga2—Sr1	84.221 (13)
As1—Sr1—Ga2^ix^	118.759 (14)	As1^viii^—Ga2—Sr1	89.655 (14)
As1^ii^—Sr1—Ga2^ix^	86.374 (9)	Ga2^viii^—Ga2—Sr1	37.413 (11)
As1^iii^—Sr1—Ga2^ix^	105.549 (10)	Sr1^xx^—Ga2—Sr1	137.673 (13)
Ga1B^iv^—Sr1—Ga2^ix^	110.30 (5)	Sr1^xxi^—Ga2—Sr1	90.483 (9)
Ga1B^v^—Sr1—Ga2^ix^	93.55 (5)	Sr1^xvii^—Ga2—Sr1	77.087 (5)
Ga1A^vi^—Sr1—Ga2^ix^	81.05 (6)	As2^viii^—Ga2—Sr1^xxii^	123.013 (14)
Ga1A^vii^—Sr1—Ga2^ix^	75.33 (5)	As1—Ga2—Sr1^xxii^	78.871 (13)
Ga2^viii^—Sr1—Ga2^ix^	154.42 (2)	As2^xvii^—Ga2—Sr1^xxii^	117.500 (15)
As2—Sr1—As2^x^	69.230 (6)	As1^viii^—Ga2—Sr1^xxii^	22.713 (10)
As2^i^—Sr1—As2^x^	150.543 (8)	Ga2^viii^—Ga2—Sr1^xxii^	37.772 (10)
As1^i^—Sr1—As2^x^	76.839 (11)	Sr1^xx^—Ga2—Sr1^xxii^	88.357 (8)
As1—Sr1—As2^x^	72.738 (10)	Sr1^xxi^—Ga2—Sr1^xxii^	111.288 (14)
As1^ii^—Sr1—As2^x^	81.367 (9)	Sr1^xvii^—Ga2—Sr1^xxii^	141.184 (8)
As1^iii^—Sr1—As2^x^	129.115 (8)	Sr1—Ga2—Sr1^xxii^	73.220 (6)
Ga1B^iv^—Sr1—As2^x^	100.60 (6)	Ga2—As1—Ga1A^xii^	114.64 (6)
Ga1B^v^—Sr1—As2^x^	140.87 (6)	Ga2—As1—Ga1B^xii^	105.91 (6)
Ga1A^vi^—Sr1—As2^x^	43.14 (7)	Ga1A^xii^—As1—Ga1B^xii^	9.02 (9)
Ga1A^vii^—Sr1—As2^x^	76.71 (7)	Ga2—As1—Ga2^viii^	73.038 (18)
Ga2^viii^—Sr1—As2^x^	42.823 (9)	Ga1A^xii^—As1—Ga2^viii^	96.32 (10)
Ga2^ix^—Sr1—As2^x^	120.222 (14)	Ga1B^xii^—As1—Ga2^viii^	96.14 (9)
As2—Sr1—As2^xi^	150.543 (8)	Ga2—As1—Ga1A^vii^	98.03 (9)
As2^i^—Sr1—As2^xi^	69.230 (6)	Ga1A^xii^—As1—Ga1A^vii^	141.90 (4)
As1^i^—Sr1—As2^xi^	72.738 (10)	Ga1B^xii^—As1—Ga1A^vii^	147.27 (17)
As1—Sr1—As2^xi^	76.839 (11)	Ga2^viii^—As1—Ga1A^vii^	112.22 (9)
As1^ii^—Sr1—As2^xi^	129.114 (8)	Ga2—As1—Sr1	128.755 (16)
As1^iii^—Sr1—As2^xi^	81.366 (9)	Ga1A^xii^—As1—Sr1	102.71 (6)
Ga1B^iv^—Sr1—As2^xi^	140.87 (6)	Ga1B^xii^—As1—Sr1	111.12 (6)
Ga1B^v^—Sr1—As2^xi^	100.60 (6)	Ga2^viii^—As1—Sr1	68.989 (12)
Ga1A^vi^—Sr1—As2^xi^	76.71 (7)	Ga1A^vii^—As1—Sr1	67.39 (9)
Ga1A^vii^—Sr1—As2^xi^	43.14 (7)	Ga2—As1—Sr1^xxi^	76.628 (13)
Ga2^viii^—Sr1—As2^xi^	120.222 (14)	Ga1A^xii^—As1—Sr1^xxi^	73.33 (8)
Ga2^ix^—Sr1—As2^xi^	42.823 (9)	Ga1B^xii^—As1—Sr1^xxi^	67.82 (8)
As2^x^—Sr1—As2^xi^	117.382 (19)	Ga2^viii^—As1—Sr1^xxi^	139.977 (16)
As1^xii^—Ga1A—As2^xiii^	114.84 (16)	Ga1A^vii^—As1—Sr1^xxi^	97.23 (9)
As1^xii^—Ga1A—Ga1B^xiv^	119.2 (2)	Sr1—As1—Sr1^xxi^	150.472 (11)
As2^xiii^—Ga1A—Ga1B^xiv^	121.43 (16)	Ga2—As1—Sr1^xvii^	65.908 (13)
As1^xii^—Ga1A—Ga1A^xiv^	127.04 (14)	Ga1A^xii^—As1—Sr1^xvii^	161.16 (10)
As2^xiii^—Ga1A—Ga1A^xiv^	112.61 (17)	Ga1B^xii^—As1—Sr1^xvii^	156.82 (8)
Ga1B^xiv^—Ga1A—Ga1A^xiv^	8.82 (9)	Ga2^viii^—As1—Sr1^xvii^	101.547 (13)
As1^xii^—Ga1A—As1^xv^	87.94 (10)	Ga1A^vii^—As1—Sr1^xvii^	32.12 (9)
As2^xiii^—Ga1A—As1^xv^	107.20 (13)	Sr1—As1—Sr1^xvii^	89.333 (9)
Ga1B^xiv^—Ga1A—As1^xv^	95.81 (13)	Sr1^xxi^—As1—Sr1^xvii^	89.018 (12)
Ga1A^xiv^—Ga1A—As1^xv^	99.49 (15)	Ga1B^xxiii^—As2—Ga2^viii^	109.16 (6)
As1^xii^—Ga1A—Sr1^xv^	151.81 (10)	Ga1B^xxiii^—As2—Ga2^xxiv^	107.80 (10)
As2^xiii^—Ga1A—Sr1^xv^	70.77 (10)	Ga2^viii^—As2—Ga2^xxiv^	111.741 (17)
Ga1B^xiv^—Ga1A—Sr1^xv^	72.42 (16)	Ga1B^xxiii^—As2—Ga1A^xxiii^	8.97 (10)
Ga1A^xiv^—Ga1A—Sr1^xv^	67.40 (7)	Ga2^viii^—As2—Ga1A^xxiii^	100.47 (6)
As1^xv^—Ga1A—Sr1^xv^	64.59 (7)	Ga2^xxiv^—As2—Ga1A^xxiii^	110.18 (10)
As1^xii^—Ga1A—Sr1^xviii^	64.22 (9)	Ga1B^xxiii^—As2—Ga1B^iv^	88.49 (12)
As2^xiii^—Ga1A—Sr1^xviii^	128.43 (8)	Ga2^viii^—As2—Ga1B^iv^	133.38 (9)
Ga1B^xiv^—Ga1A—Sr1^xviii^	63.92 (16)	Ga2^xxiv^—As2—Ga1B^iv^	102.40 (9)
Ga1A^xiv^—Ga1A—Sr1^xviii^	68.55 (7)	Ga1A^xxiii^—As2—Ga1B^iv^	96.31 (10)
As1^xv^—Ga1A—Sr1^xviii^	123.83 (13)	Ga1B^xxiii^—As2—Sr1	127.93 (10)
Sr1^xv^—Ga1A—Sr1^xviii^	135.95 (13)	Ga2^viii^—As2—Sr1	70.223 (14)
As1^xii^—Ga1A—Sr1^xix^	44.97 (5)	Ga2^xxiv^—As2—Sr1	120.879 (15)
As2^xiii^—Ga1A—Sr1^xix^	105.82 (11)	Ga1A^xxiii^—As2—Sr1	128.03 (10)
Ga1B^xiv^—Ga1A—Sr1^xix^	127.80 (7)	Ga1B^iv^—As2—Sr1	65.20 (9)
Ga1A^xiv^—Ga1A—Sr1^xix^	135.48 (17)	Ga1B^xxiii^—As2—Sr1^xxii^	71.67 (8)
As1^xv^—Ga1A—Sr1^xix^	46.51 (6)	Ga2^viii^—As2—Sr1^xxii^	73.974 (13)
Sr1^xv^—Ga1A—Sr1^xix^	107.02 (6)	Ga2^xxiv^—As2—Sr1^xxii^	66.325 (12)
Sr1^xviii^—Ga1A—Sr1^xix^	104.00 (10)	Ga1A^xxiii^—As2—Sr1^xxii^	66.09 (8)
As1^xii^—Ga1A—Sr1^xiii^	85.55 (10)	Ga1B^iv^—As2—Sr1^xxii^	151.44 (9)
As2^xiii^—Ga1A—Sr1^xiii^	29.68 (6)	Sr1—As2—Sr1^xxii^	143.341 (12)
Ga1B^xiv^—Ga1A—Sr1^xiii^	149.64 (10)	Ga1B^xxiii^—As2—Sr1^xxv^	21.56 (6)
Ga1A^xiv^—Ga1A—Sr1^xiii^	140.99 (16)	Ga2^viii^—As2—Sr1^xxv^	128.411 (14)
As1^xv^—Ga1A—Sr1^xiii^	102.85 (10)	Ga2^xxiv^—As2—Sr1^xxv^	103.930 (14)
Sr1^xv^—Ga1A—Sr1^xiii^	94.16 (9)	Ga1A^xxiii^—As2—Sr1^xxv^	30.44 (6)
Sr1^xviii^—Ga1A—Sr1^xiii^	120.80 (7)	Ga1B^iv^—As2—Sr1^xxv^	68.27 (8)
Sr1^xix^—Ga1A—Sr1^xiii^	81.80 (6)	Sr1—As2—Sr1^xxv^	120.158 (9)
As2^xiii^—Ga1B—As1^xii^	116.66 (16)	Sr1^xxii^—As2—Sr1^xxv^	88.418 (8)
As2^xiii^—Ga1B—Ga1B^xiv^	125.23 (17)	Ga1B^xxiii^—As2—Sr1^xxvi^	139.37 (9)
As1^xii^—Ga1B—Ga1B^xiv^	117.83 (16)	Ga2^viii^—As2—Sr1^xxvi^	97.627 (13)
As2^xiii^—Ga1B—Ga1A^xiv^	116.7 (2)	Ga2^xxiv^—As2—Sr1^xxvi^	32.041 (11)
As1^xii^—Ga1B—Ga1A^xiv^	126.56 (18)	Ga1A^xxiii^—As2—Sr1^xxvi^	142.22 (10)
Ga1B^xiv^—Ga1B—Ga1A^xiv^	8.90 (9)	Ga1B^iv^—As2—Sr1^xxvi^	94.88 (8)
As2^xiii^—Ga1B—As2^xvi^	80.20 (9)	Sr1—As2—Sr1^xxvi^	89.306 (7)
As1^xii^—Ga1B—As2^xvi^	102.75 (11)	Sr1^xxii^—As2—Sr1^xxvi^	87.812 (8)
Ga1B^xiv^—Ga1B—As2^xvi^	93.10 (15)	Sr1^xxv^—As2—Sr1^xxvi^	130.398 (9)

Symmetry codes: (i) *x*−*y*, −*y*, −*z*+1/3; (ii) *x*, *y*−1, *z*; (iii) *x*−*y*+1, −*y*+1, −*z*+1/3; (iv) *y*, *x*−1, −*z*+1; (v) −*x*+*y*+1, −*x*+1, *z*−2/3; (vi) *y*−1, *x*−1, −*z*+1; (vii) −*x*+*y*, −*x*+1, *z*−2/3; (viii) *y*, *x*, −*z*; (ix) −*x*+*y*, −*x*, *z*+1/3; (x) *y*, *x*−1, −*z*; (xi) −*x*+*y*+1, −*x*+1, *z*+1/3; (xii) *y*, *x*, −*z*+1; (xiii) *x*, *y*+1, *z*+1; (xiv) −*x*+2, −*x*+*y*+1, −*z*+5/3; (xv) −*y*+1, *x*−*y*+1, *z*+2/3; (xvi) *y*+1, *x*, −*z*+1; (xvii) *x*−1, *y*, *z*; (xviii) −*y*+1, *x*−*y*, *z*+2/3; (xix) −*y*, *x*−*y*, *z*+2/3; (xx) −*y*, *x*−*y*, *z*−1/3; (xxi) *x*, *y*+1, *z*; (xxii) −*y*+1, *x*−*y*, *z*−1/3; (xxiii) *x*, *y*−1, *z*−1; (xxiv) *x*+1, *y*, *z*; (xxv) −*y*+1, *x*−*y*−1, *z*−1/3; (xxvi) *x*+1, *y*+1, *z*.
